# Shipping routes through core habitat of endangered sperm whales along the Hellenic Trench, Greece: Can we reduce collision risks?

**DOI:** 10.1371/journal.pone.0212016

**Published:** 2019-02-27

**Authors:** Alexandros Frantzis, Russell Leaper, Paraskevi Alexiadou, Aristides Prospathopoulos, Dimitrios Lekkas

**Affiliations:** 1 Pelagos Cetacean Research Institute, Vouliagmeni, Greece; 2 International Fund for Animal Welfare, London, United Kingdom; 3 Hellenic Centre for Marine Research, Institute of Oceanography, Anavyssos, Greece; 4 MarineTraffic Operations SA, Athens, Greece; Aristotle University of Thessaloniki, GREECE

## Abstract

The Mediterranean sperm whale population is listed as ‘Endangered”. The Hellenic Trench is the core habitat of the eastern Mediterranean sperm whale sub-population that numbers two to three hundred individuals. Major shipping routes running on or very close to the 1000 m depth contour along the Hellenic Trench are causing an unsustainable number of ship-strikes with sperm whales reviewed in this paper. Sperm whale sighting and density data were combined with specific information on the vessel traffic in the area (e.g., types of vessels, traffic patterns, speed and traffic density), in order to estimate the risk of a whale/ship interaction. Routing options to significantly reduce ship strike risk by a small offshore shift in shipping routes were identified. The overall collision risk for sperm whales in the study area would be reduced by around 70%, while a maximum of 11 nautical miles would be added to major routes and only around 5 nautical miles for the majority of ships. No negative impacts were associated with re-routing by shipping away from sperm whale habitat and there would be additional shipping safety and environmental benefits. A significant contribution to the overall conservation status of the marine Natura2000 sites in the area and very important population units of threatened species such as Cuvier’s beaked whales, monk seals and loggerhead turtles would be achieved, by the reduction of shipping noise and reduced risk of any oil spills reaching the coasts, which are also important touristic destinations in Greece.

## Introduction

The Hellenic Trench is core habitat for the eastern Mediterranean sperm whale sub-population, which is believed to number no more than two to three hundred individuals [1, 2]. The Mediterranean sperm whale population is listed as “Endangered” by International Union for the Conservation of Nature (IUCN) and is at risk mainly from driftnet entanglement and ship strikes [3]. Because of its regional importance for the Mediterranean sperm whale population, ACCOBAMS (Agreement on the Conservation of Cetaceans of the Black Sea, Mediterranean Sea and contiguous Atlantic area) proposed the Hellenic Trench to become a Marine Protected Areas for cetaceans [4]. More recently, under the title “Hellenic Trench, Deep Diver's Sanctuary”, this sea area received the status of Important Marine Mammal Area (IMMA) at a global scale [5].

The most recent estimate for the total population of sperm whales in the Eastern Mediterranean is 164 [2]. This estimate comes from line-transect surveys covering different areas in 2003, 2007 and 2013 generating a total estimate of 147 for the surveyed areas with a 95% confidence interval of 74–289. Extrapolation to unsurveyed areas suggested a total of 164 but with a number of caveats. These estimates include a correction for g(0) (the probability that a whale directly on the trackline will be detected) but do not include young calves who may not be vocalising [2]. A photo-identification study from the Hellenic Trench, also resulted in 164 distinct individuals photo-identified from 1998 to 2009 if those that were known to have died were excluded [1]. The slope on the discovery curve indicated that most animals sighted in the last years of the study were already known, with a small portion of the population remaining to be discovered. This study included matches with other areas, suggesting that the Hellenic Trench area is used by the entire population within the Eastern Mediterranean [1]. For example, one of the three sperm whales photographed SW of Paphos in a recent survey off Cyprus in 2017 had already been photo-identified south of Zakynthos Island along the Hellenic Trench in the Ionian Sea in 2008 [6]. All this evidence leads to the assumption of a total population of roughly 200–300 individuals for both the Hellenic Trench [1] and the entire eastern Mediterranean basin.

Frantzis et al. [1] found a pronounced peak in sperm whale density along the Hellenic Trench around the 1000 m depth contour. Estimated density decreased in both shallower and deeper waters, with 74% of the total within 3 km of the 1000 m contour. As in other areas, shipping follows direct routes around land masses keeping a safe distance from shore. In the west and southwest Hellenic Trench this results in major shipping routes running often on or very close to the 1000 m depth contour, concentrating shipping and sperm whales within the same small area. Based on an analysis of sperm whale and shipping distribution patterns in the Hellenic Trench [7, 8], which noted the potential for small changes in shipping routes to dramatically reduce risk, the Scientific Committee of the International Whaling Commission (IWC) recommended that interested parties (including Greece, ACCOBAMS and the shipping industry) move forward in order to jointly develop a proposal for routing measures in accordance with the International Maritime Organization (IMO) guidelines [9, 10].

The above described situation in the Hellenic Trench was brought to the attention of the Marine Environment Protection Committee (MEPC) of the IMO in 2016. The IMO welcomed the focus on the separation of ships and cetaceans in areas where the latter congregate and noted that minor routing changes in high risk areas was possibly the best measure for reducing ship strikes and could lead to substantial reduction in strikes [11]. The IWC had listed the Hellenic Trench among four ship-strike areas of particular concern at the global level [11]. The IMO had previously adopted a guidance document for minimizing the risk of ship strikes with cetaceans in 2009 [12], which lists a number of principles to be taken into account by proposals for action to reduce ship strikes.

This paper aims to address the problem of ship strikes along the Hellenic Trench by (i) documenting the available research on sperm whales as well as specific information on the vessel traffic in the area (e.g., types of vessels, traffic patterns, and traffic density), such that the risk of a whale/ship interaction can be estimated, (ii) identifying possible routing options to reduce ship strike risk and (iii) assessing the implications of the proposed routing options with respect to other species vulnerable to shipping impacts and fishing activities

## Methods

### Sperm whale distribution data and density analysis

**Fig 1:**
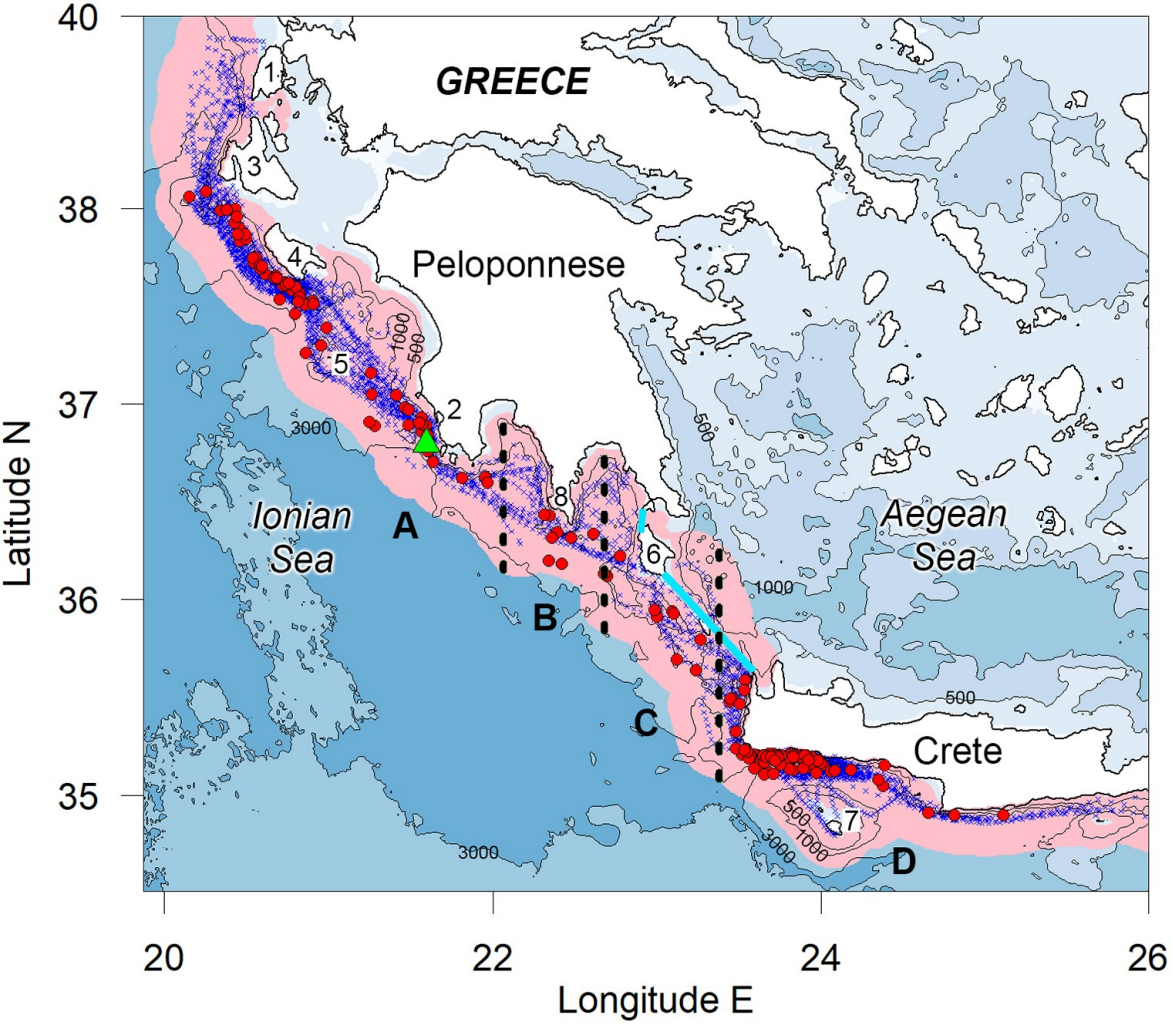
**Acoustic listening stations (blue dots), visual sightings of sperm whales (red dots) and study zones.** The study zones (A, B, C, D) are delimited by black doted lines. The radius of the circles around the acoustic listening stations (overlapping pink circles) is 16.9 km, representing the estimated effective radius of detection for the mean sperm whale group size. The green triangle indicates the position of the fixed acoustic recording station Poseidon-Pylos. The cyan line indicates the limits of the Ionian Sea that we considered for estimating mean shipping density. The study zones are: Zakynthos Island and west Peloponnese (A), Cape Tainaron (B), Kythira Sea (C), Crete (D). Numbers indicate Greek locations mentioned in the text as follows. 1: Lefkada Island, 2: Pylos, 3: Kefallonia Island, 4: Zakynthos Island, 5: Strofades Islands, 6: Kythira Island, 7: Gavdos Island, 8: Cape Tainaron.

All research with sperm whales was conducted under the Research Permit by the Ministry of Environment & Energy (Greece) Number 152931/539/2017. Surveys along the Hellenic Trench were conducted yearly between 1998 and 2009 and in 2014, 2015 and 2017 from June to October, with the majority of survey effort in July and August. The study area encompassed the west and southwest part of the Hellenic Trench from Lefkada Island (Location 1 on Fig 1) to south Crete (Fig 1). A towed hydrophone was monitored every fifteen minutes for sperm whale vocalisations and each listening station was assigned a binary status as either ‘sperm whales detected’ or ‘no sperm whales detected’. When sperm whales were detected, the effort switched from searching to tracking and acoustic bearings were used to direct the vessel towards the individual or group for photo-identification and behavioural observations. The vessel was considered to be ‘on acoustic effort’ when on a predetermined trackline until the first acoustic detection was made. It was considered to be ‘off effort’ while tracking detected whales and during visual encounters. At the end of an encounter with a whale or a group of whales (which could extend over many hours) the vessel steamed away for at least 5 km before acoustic monitoring effort resumed. Furthermore, while leaving a whale or whale group and until reaching 20 km of distance, any detection that had a bearing coming from behind the vessel was not considered. In addition, no sea area was searched acoustically more than once during the same day, so no acoustic effort was considered when a second passage occurred (e.g. when returning to the port of departure on the same day using the same route in the opposite direction). A detailed description of the combined acoustic searching and visual observation methods is given in Frantzis et al. [1]. Estimates of the detection range at which whales could be heard were made from 62 experiments during which the research vessel travelled directly away from a vocalising individual or group. This gave estimates of the effective detection range of the system (the distance at which there is an equal probability of missing a whale within that distance or detecting a whale at a greater distance) of 10.7 to 21.1 km for different sizes and types of sperm whale groups [Gkikopoulou, pers. comm.]. The effective radius of detection of 16.9 km for the ‘mean sperm whale group size’ was used for this study. This detection radius is comparable with the 15 km detection radius for sea state 3 in André et al [13] and 16 km for ‘usual click’ from males in Madsen et al [14]. It is higher than typical perpendicular effective strip widths from acoustic surveys (e.g. 10 km from [15]), which have higher flow noise associated with the speed of the vessel.

In order to evaluate the changes in ship routes as possible mitigation options for ship strikes, the average relative density of whales along the current route was compared to possible alternatives. The approach used for this analysis was to use the locations of all sperm whale sightings together with data on shipping density to identify high risk areas. A simple index of relative density for each of the areas in Fig 1 was calculated as the proportion of days with acoustic search effort in the area on which whales are encountered.

### Sperm whale presence outside of summer survey periods

In order to investigate the presence of sperm whales out of the survey period (June to October), we (i) examined all available sightings of sperm whales either opportunistic or originating from independent dedicated surveys along the Hellenic Trench and (ii) analyzed acoustic data from a fixed acoustic recording station positioned approximately at the middle of the latitudinal extents of the study area, off Pylos (36.8° N, 21.6° E, see Fig 1), at a depth of 500 m. The sea bottom depth for this location is 1680 m.

Opportunistic sightings originated from the national database of cetacean sightings maintained by the Pelagos Cetacean Research Institute (PCRI). The database contains 1963 records of independent cetacean sightings of identified species originating from either opportunistic observations documented with visual material (photographs or videos) or sightings during dedicated cetacean surveys. Thirty five sightings concern the historical period 1974–1991 and 1928 sightings concern the period 1992–2017.

The acoustic recordings were made by a Passive Acoustic Listener (PAL) with a sampling rate of 100 kHz. This specific device was deployed in two periods from 12 November 2008 to 17 September 2009 and from 29 November 2009 to 6 July 2010. The PAL was attached on the permanent mooring of the oceanographic buoy Poseidon-Pylos of the Poseidon Monitor Forecasting and Information System for the Greek Seas (http://poseidon.hcmr.gr). PAL recordings lasted 2.8 s and 4.5 s and had an average total duration of 0 h 55 min and 3 h 27 min per day for the sampling periods 2008–2009 and 2009–2010, respectively. More details regarding the Poseidon and PAL systems are given by Nystuen et al. [16].

PAL recordings were examined aurally by the first author. Only unequivocal sperm whale detections of characteristic regular clicks or codas were kept for statistical analysis. Each day of recordings was classified as “day with/without” sperm whales. Multiple detections in consecutive or different recordings of one day had no more weight than a single detection in another day.

### Direct evidence of collisions

The cetacean stranding database maintained by the PCRI was used to investigate the presence of stranded sperm whales with signs of a collision with a large vessel. The database, which concerns all the Greek territory, covers (i) historical strandings up to 1991 with 104 available records and (ii) 2451 more recent strandings collected systematically during the last 25 years from 1992 up to 2017. The vast majority of stranding reports arrive directly to PCRI from the Greek Port-Police authorities and mainly include date, location, photographs and total length of the stranded animal. Additional stranding records are collected from local volunteers all around Greece, or citizens who report strandings through an internet stranding form. Whenever possible, *in situ* inspections, full measurements, necropsies and sample collection or skeleton recovery are taking place.

In addition, sperm whale photo-identification data of free-ranging sperm whales from surveys (see [1]) were examined for animals showing scars or wounds that could be attributed to ship strikes.

### Ship density estimates

Ship density estimates were based on data from Automatic Identification System (AIS) transmissions received from both satellite and terrestrial receivers of the Marine Traffic network (www.marinetraffic.com) for one year (31 July 2015 to 31 July 2016). Shipping density was defined according to Leaper and Panigada [17] as the distance travelled in km per km^2^ (i.e. the units are km^-1^). Data were filtered such that if the same transmission signal was received on both satellite and terrestrial receivers, it was recorded as terrestrial. When a vessel is within range of a terrestrial receiver then transmissions are received frequently (every few seconds) and the complete track of the vessel can be established. In these situations the contribution of that vessel transit to the density in each grid square was the reported speed over ground (SOG) multiplied by the time interval for all transmissions with the location in that grid square. As vessels move away from the coast, the receptions on terrestrial aerials become more intermittent. These are then supplemented by satellite data. Satellites only receive for the duration that they are above the horizon from a particular location.

To obtain an unbiased estimate of shipping density for a grid square let *t* be the time interval between the previous satellite pass and the pass where a signal is received in that square.

The distance *D* allocated to the grid square is then given by *D* = *vt*, where *v* is the vessel speed

If *L* is the true length of track within the square and the satellite pass is considered as instantaneous then the probability *p* that a ship will be recorded given the time interval *t* is given as:

$$p=P(\mathrm{Recorded}|t)=\frac{L}{vt},$$ (1)

and the expected contribution to the density estimate will be equal to *pD*. Consequently, as it can be seen from Eq (1) the resulting contribution to the estimate of shipping density will be *L*.

For the offshore areas, more than 200 km far from the coast, almost all the data is derived from satellite signals. To detect whether there was any bias between coastal and offshore estimates, density estimates along a shipping route with little evidence of crossing traffic were examined. For the route heading approximately east-west around latitude 36.5^o^, the average densities across a number of 30 km by 1 km rectangles with the longer side perpendicular to the route were compared.

AIS data from fishing vessels were also analysed in order to examine any implications of ship routing for fishing activities. Since May 2014, it has been mandatory for all EU fishing vessels over 15 m to be equipped with AIS. Fishing presence was measured in terms of time within an area rather than distance travelled, because this more accurately reflects the potential for interactions with other shipping.

To estimate the mean shipping density at the locations of sperm whale encounters, we used the annual average for each one km grid square that encompassed at least one sperm whale sighting position. Shipping density was expressed in km^-1^year^-1^.

### Collision risk estimates

The risk of contact between a vessel and a whale will be a function of the number of collisions that would occur if no avoiding action was taken by the whale or the vessel, multiplied by the proportion of occasions when any response successfully avoids contact. Very little is known about sperm whale responses to vessels but an index of relative risk within an area was assumed to be proportional to whale density multiplied by shipping density. This index of relative risk was used to determine the risk reduction that might be achieved by alternative routing options. An alternative would be to weight the shipping density according to vessel speed using a derived speed-risk relationship such as that in [18]; however, since the routing options considered were assumed to apply to all large vessels, the relative risk reduction achieved would be independent of vessel speed. To examine collision risk and routing options, the study region was divided into four zones depicted in Fig 1: west of Zakynthos Island and the Peloponnese down to 36.5^o^N (A); south of Cape Tainaron (B); between Kythira Island and Crete (C); waters around Crete (D).

For each zone, the collision risk index *C* was defined as

$$C=W_{d}\times G\times D_{SW},$$ (2)

where *W*_*d*_ is the proportion of survey days with whales present, *G* is the mean sperm whale group size within that zone and *D*_*SW*_ is the average of the average annual shipping density across all locations within a zone where whales were observed (i.e. the collision risk index is an index of whale density multiplied by the average exposure of each whale to shipping). For alternative routing measures, future *D*_*SW*_ was estimated assuming total avoidance of specified areas.

## Results

### Sperm whale distribution

The data set comprised a total of 4606 listening stations. Surveys were conducted with a total of 385 survey days and more than 17,000 km of on effort trackline. Between 1998 and 2009, 4399 listening stations resulted in 178 visual encounters with sperm whales (see [1]). 207 additional listening stations from surveys in the years 2014, 2015 and 2017 resulted in eight more visual encounters. Search effort was conducted from June to October but with the majority in July and August (Table 1). The proportion of listening stations with sperm whale detections suggests consistent use of the area by sperm whales between June-September but significantly less use in October (Binomial test, p = 0.03) although the search effort in October was relatively small.

**Table 1:** Summary of acoustic search effort and sperm whale detections.

	1998–2009		2014, 2015, 2017	
Month	Number of listening stations	Number of listening stations where sperm whales were heard	Number of listening stations	Number of listening stations where sperm whales were heard
June	71	6	13	3
July	1835	107	94	3
August	2221	110	100	1
September	200	15		
October	73	0		

The distribution of acoustic search effort and location of sperm whale sightings are shown in Fig 1. In most cases, the acoustic search effort extends considerably further offshore than the sighting locations. Hence, it is unlikely that there were significant numbers of animals further offshore that were not detected.

### Sperm whale presence outside of summer survey periods

**Fig 2:**
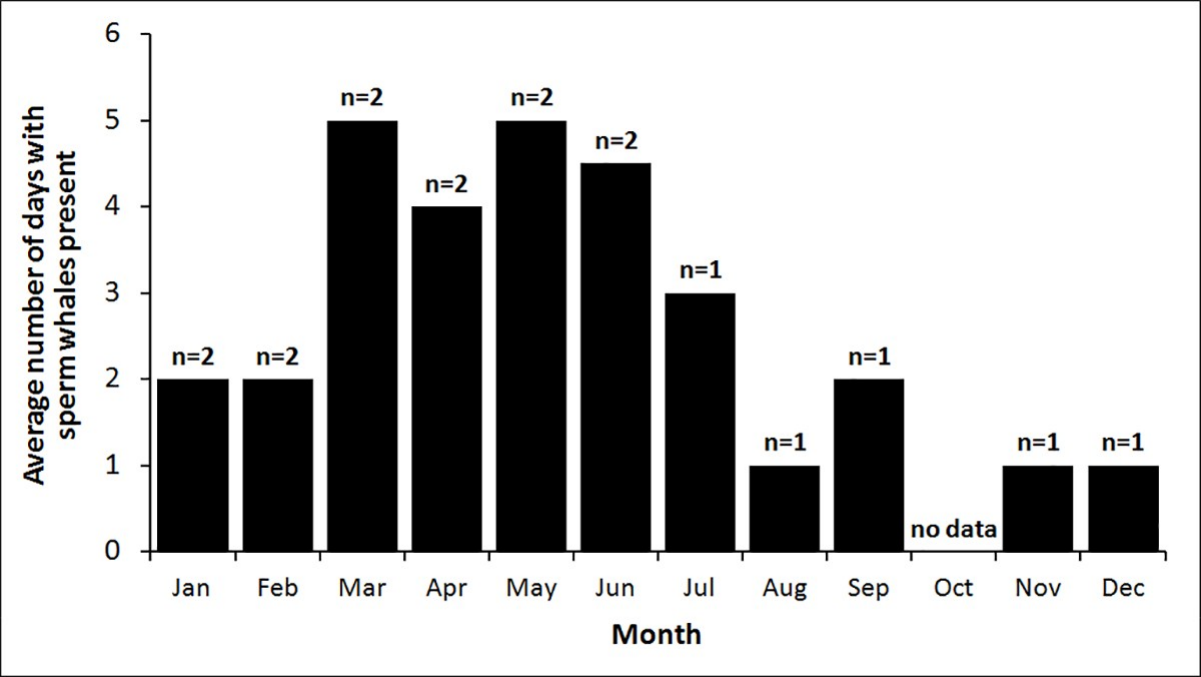
Data on year round presence of sperm whales along the Hellenic Trench. Average number of days with sperm whales detected on the fixed PAL acoustic recorder. Values of n indicate the number of years with data for each month.

The fixed acoustic recording station showed sperm whale presence in all months of the year except October, for which no data were available (Fig 2).

According to the results obtained by the fixed acoustic station, it appears that sperm whale presence is greater from March-July (ANOVA, p<0.01), but this needs to be treated with caution because of the small sample sizes (1–2 years) and of the monitoring at a single site. The mean number of days per month with sperm whales present in July and August was 2, which is slightly lower than the overall average of 3.1 days per month and the average of 3 days per month for June-September. However, these differences are not significant. Most of the dedicated survey effort (92% of listening stations) was conducted in July and August with 8% spread across June, September and October. Based on the limited year round data there is no reason to believe that the dedicated surveys would have encountered substantially different numbers of sperm whales than the average presence throughout the year.

Of 328 sperm whale sightings in the database maintained by PCRI, eighty two that originated from sources independent to our surveys were made within the study area limits along the Hellenic Trench (Fig 1). These data show sperm whale presence in the study area in all months of the year except December (for which presence was detected from the fixed acoustic recorder). Presence through sightings includes the month of October for which no data were available from the fixed acoustic recorder (PAL). The number of sightings per month cannot be weighted by effort, which was much higher in summer months for opportunistic sighting contributing sources due to summer vacations, tourism, dolphin and whale watching operations and better weather conditions for sightings. In contrast, very few people are out in the sea to report any sighting during the winter months.

### Direct evidence of collisions

**Fig 3:**
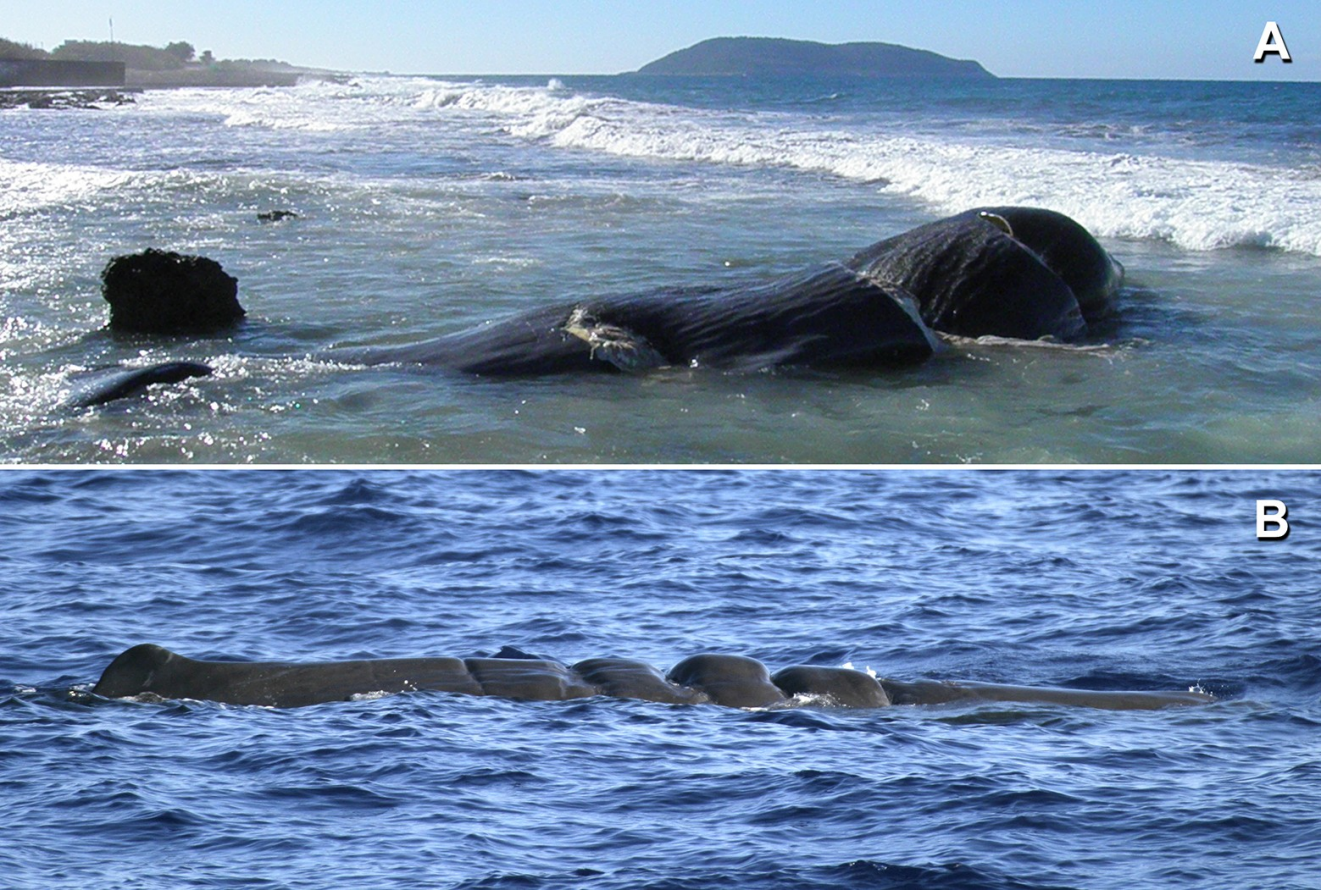
Propeller marks on stranded and free ranging sperm whales along the Hellenic Trench. (A) One of the recent cases of ship strike with a sperm whale that occurred along the Hellenic Trench on 15 February 2014, 25 km north of Pylos in SW Peloponnese. Three big cuts from a large propeller were apparent on this very freshly dead whale. (B) One of the live sperm whales photographed in 2004 along the Hellenic trench in SW Crete (catalogued with the name “Ifaistos”) bearing clear and deep marks from a ship propeller between the head and the dorsal fin. Photo credits (A): Filiatranet, (B): Chris Johnson.

Thirty strandings of sperm whales have been recorded from 1992 to 2017 along the Greek coasts. In twenty four of these cases there was a visit *in situ* or the available photographic material that was collected shows the animal soon after the stranding. Thirteen cases concern sperm whales with propeller marks or cuts that only a ship strike could make (Table 2, Fig 3A). Three more cases could be the result of a ship strike and eight cases had no obvious ship strike signs or the death was shown to have another cause. For the remaining six cases the cause of death was unknown. Six of the stranded sperm whales that had ship strike marks plus three that potentially could be the result of a ship strike were found in the area of the Hellenic Trench. The total number of ship strikes with sperm whales is not known, since it would be expected that a number of carcasses would not strand or otherwise be recorded. In addition, at least four out of 181 photo-identified sperm whales during surveys along the Hellenic Trench (reported in [1]) had deep wounds apparently caused by propellers (Fig 3B).

**Table 2:** Data on sperm whale strandings that occurred along the coasts neighboring the Hellenic Trench (HT) or other Greek coasts from 1992 to 2017 and had marks from a ship strike or could potentially be due to a ship strike.

#	Year	Location	Notes
1	1997	East Aegean Sea	Very fresh animal. Missing tailstock and large cut behind the dorsal fin.
2	1997	Cyclades, Aegean Sea	Three deep propeller cuts in front of the dorsal fin.
3	2001	SW Crete, HT	Very fresh animal. Large open wound at the base of the skull.
4	2001	Cyclades, Aegean Sea	2 m long scission laterally, from lower jaw to pectoral fin, potentially caused by a ship strike.
5	2001	Cyclades, Aegean Sea	Three propeller cuts, two in front and one behind the dorsal fin.
6	2002	W Peloponnese, HT	Decomposed animal cut in two pieces, potentially cut after a ship strike
7	2004	North Crete	Two deep propeller marks laterally and ventrally, in front and behind the pectoral fin.
8	2005	W Peloponnese, HT	Three propeller cuts or marks, two in front and one behind the pectoral fin
9	2006	NW Aegean Sea	Two deep propeller cuts at the front and above and the blowhole
10	2007	West Crete, HT	Lateral hit at the base of skull; stranded three days before the next record in the same location
11	2007	West Crete, HT	Fresh animal cut in two just behind the dorsal fin.
12	2010	Central-East Aegean Sea	Very fresh animal with deep cut dorsally at the level of the blowhole.
13	2011	Corfu, North HT	Fresh animal. Cut and missing tailstock.
14	2012	Kythira Island, HT	Decomposed animal with cut at the tailstock potentially caused by a ship strike.
15	2014	SW Peloponnese, HT	Three deep propeller cuts, two in front and one behind the dorsal fin.
16	2016	SE Peloponnese, HT	Four deep propeller cuts, all along the body.

### Shipping density and characterisation of ship traffic

Comparisons with earlier analysed AIS data (2012–13; see [7]) showed consistent patterns of shipping over time with no obvious changes in the routes. Hence, just the most recent data were used for the risk analysis.

**Fig 4:**
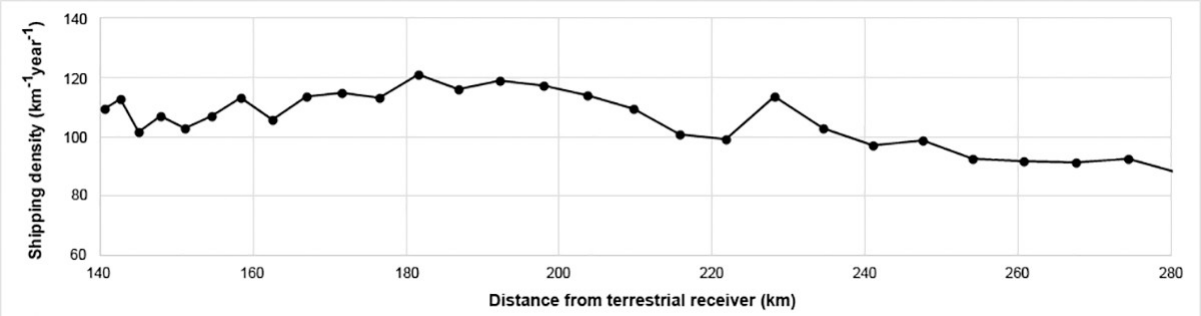
Average shipping density estimates against distance from terrestrial receiver on Zakynthos Island.

Density estimates along a sample route are shown in Fig 4 against distance from the predominant receiving station (Zakynthos Island, 37.73^o^ N, 20.87^o^ E, 500 m elevation). Received signals comprised of a mixture of terrestrial and satellite data with predominantly satellite data at distances greater than 200 km and terrestrial data at smaller distances.

Data across the whole route for traffic heading approximately east-west around 36.5^o^ N are shown.Some variability in the density estimates is visible along the route, but there is no evidence of substantial relative bias between satellite and terrestrial data.

**Fig 5:**
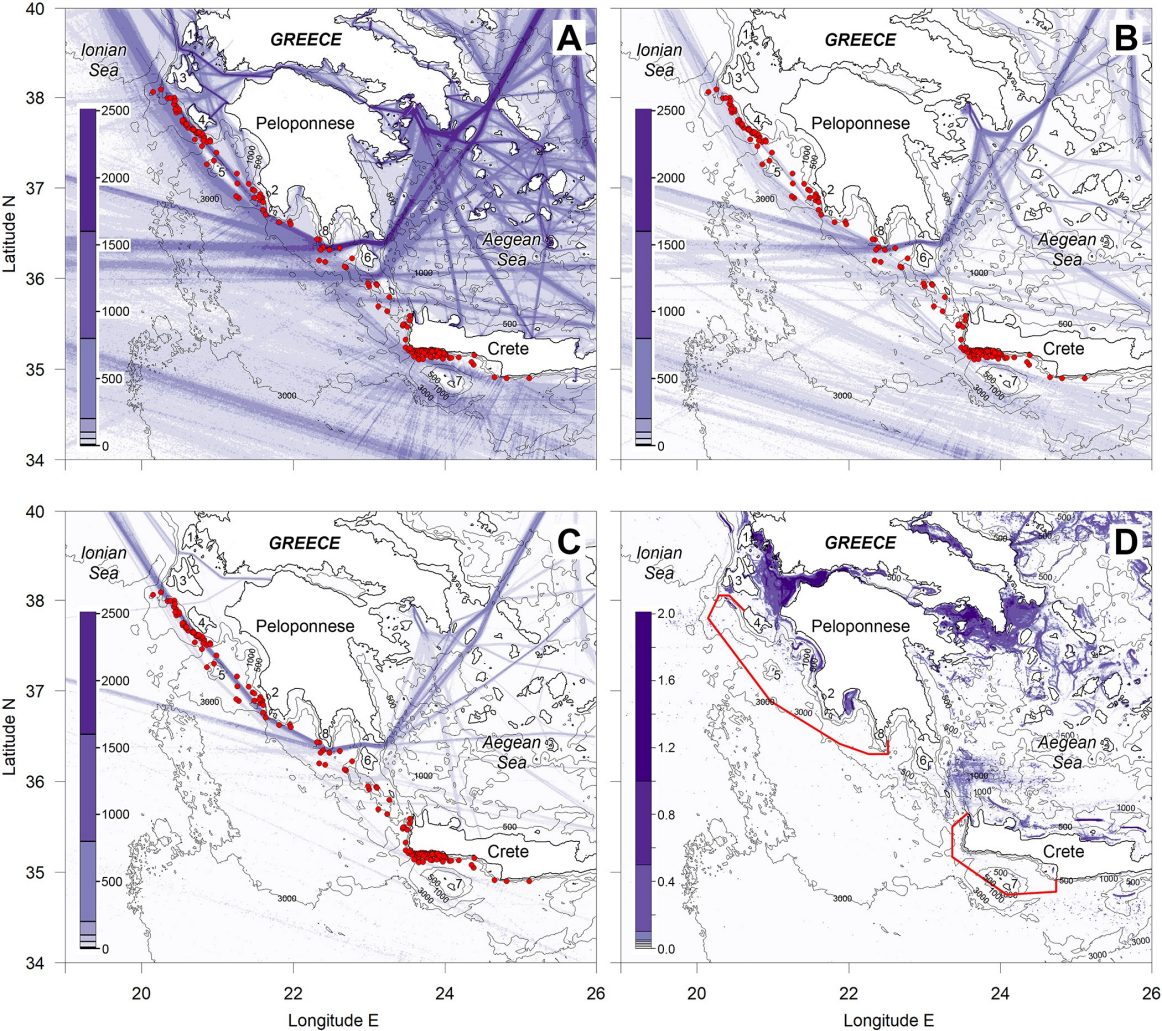
Density of ship traffic reported through AIS transmissions and sperm whale sightings (red dots) from our surveys. Opportunistic and independent survey sightings are not presented. (A) Density of all ship traffic, excluding fishing vessels. (B) Density of container ship traffic. Traffic to the SW of Crete north of Gavdos Island is mainly dominated by container ships heading to or from the eastern Mediterranean (ports to the east of Port Said and the Suez Canal) through the Strait of Otranto or Strait of Messina. (C) Density of Ro-Ro cargo traffic. The main route of concern for sperm whales is between the Strait of Otranto and the Aegean via Elafonisos Strait, north of Kythira Island. (D): Fishing vessel presence by time spent in each grid square. The scale for shipping density plots (A), (B) and (C) is in km^-1^year^-1^ and for plot (D) is in days.km^-2^.year^-1^. Numbers indicate Greek locations mentioned in the text as follows. 1: Lefkada Island, 2: Pylos, 3: Kefallonia Island, 4: Zakynthos Island, 5: Strofades Islands, 6: Kythira Island, 7: Gavdos Island, 8: Cape Tainaron.

Fig 5A shows the major shipping routes and the clear overlap with sperm whale encounters within the study area. For the areas of potential sperm whale habitat (water depth > 500 m) in Fig 5A outside of the Aegean Sea (i.e. west of a line between Kythira Island and NW Crete; see Fig 1), the mean shipping density was 70 km^-1^year^-1^. The mean shipping density at the locations of sperm whale encounters was 220 km^-1^year^-1^ (sd = 313 km^-1^year^-1^) ranging from 0 to 2291 km^-1^year^-1^.

For the area of concern to the west of the Peloponnese, shipping heading between the Adriatic through Strait of Otranto and the Aegean passes either to the east or west of Strofades Islands (Location 5 on Fig 5) before merging again into a single route west of Kefallonia Island (Location 3 on Fig 5). This merged traffic is primarily made up of vessel types presented in Table 3. The traffic is dominated by general cargo vessels (24%, Fig 5B), with the average speed of these vessels around 10 knots. However, Ro-Ro cargos (Fig 5C), which accounted for 14% of the traffic, had a much greater average speed (18 knots). The same vessels tend to operate on this route many times a year with a single Ro-Ro vessel doing 104 transits in the year (twice weekly). Other categories of relatively fast vessels on this route included 1033 transits of container ships (with an average speed of 14.8 knots). 68% of these transits were by vessels that travelled at least 12 times a year (i.e. once a month) with a single vessel having made 69 transits in the year. Large passenger cruise ships also travel relatively fast and accounted for 1251 transits in the year (Table 3).

**Table 3:** Composition and statistics on traffic passing west of Kefallonia Island while heading to and from the Strait of Otranto.

Vessel type	Number of transits in one year	Proportion	Average SOG (knots)
General Cargo	3162	0.24	10.1
Ro-Ro Cargo	1812	0.14	18.4
Bulk Carrier	1414	0.11	12.0
Passenger Ship	1251	0.09	13.4
Container Ship	1033	0.08	14.8
**Total**	**13192**	**0.66**	**12.5**

Most of the Ro-Ro cargo traffic (99% of the total on the north-south route) heading to or from the Adriatic passes east of Strofades Islands (Location 5 on Fig 5) compared to 28% of container ships and 19% of passenger ships. This route is slightly shorter (by around 5 nautical miles) than the main route west of Strofades Islands and tends to be used by the faster vessels. In particular, passenger vessels choosing to pass east of Strofades Islands had an average speed of 17.4 knots (Table 4) compared to the overall average for passenger vessels of 13.4 knots. The passage east of Strofades Islands runs directly along the 1000 m contour in a number of places and particularly for around 50 nm west of Zakynthos Island (Location 4 on Fig 5), which is an area of great importance for sperm whales [1]. However, around 60% of the traffic already chooses to pass west of Strofades Islands, presumably due to navigational safety considerations.

**Table 4:** Composition and statistics on traffic passing east of Strofades Islands before merging with traffic to pass west of Kefallonia Island while heading to and from the Strait of Otranto.

Vessel type	Number of transits in one year	Proportion	Average of SOG (knots)
Ro-Ro Cargo	1792	0.36	18.6
General Cargo	1489	0.30	9.8
Bulk Carrier	446	0.09	11.8
Container Ship	287	0.06	14.5
Passenger Ship	234	0.05	17.4
Vehicles Carrier	160	0.03	16.0
**Total**	**4948**	**0.89**	**14.2**

Total fishing vessel presence in terms of days at sea per year per km^2^ is shown in Fig 5D for all fishing vessels equipped with AIS (mandatory for all EU fishing vessels over 15 m).

### Options to reduce risk

**Fig 6:**
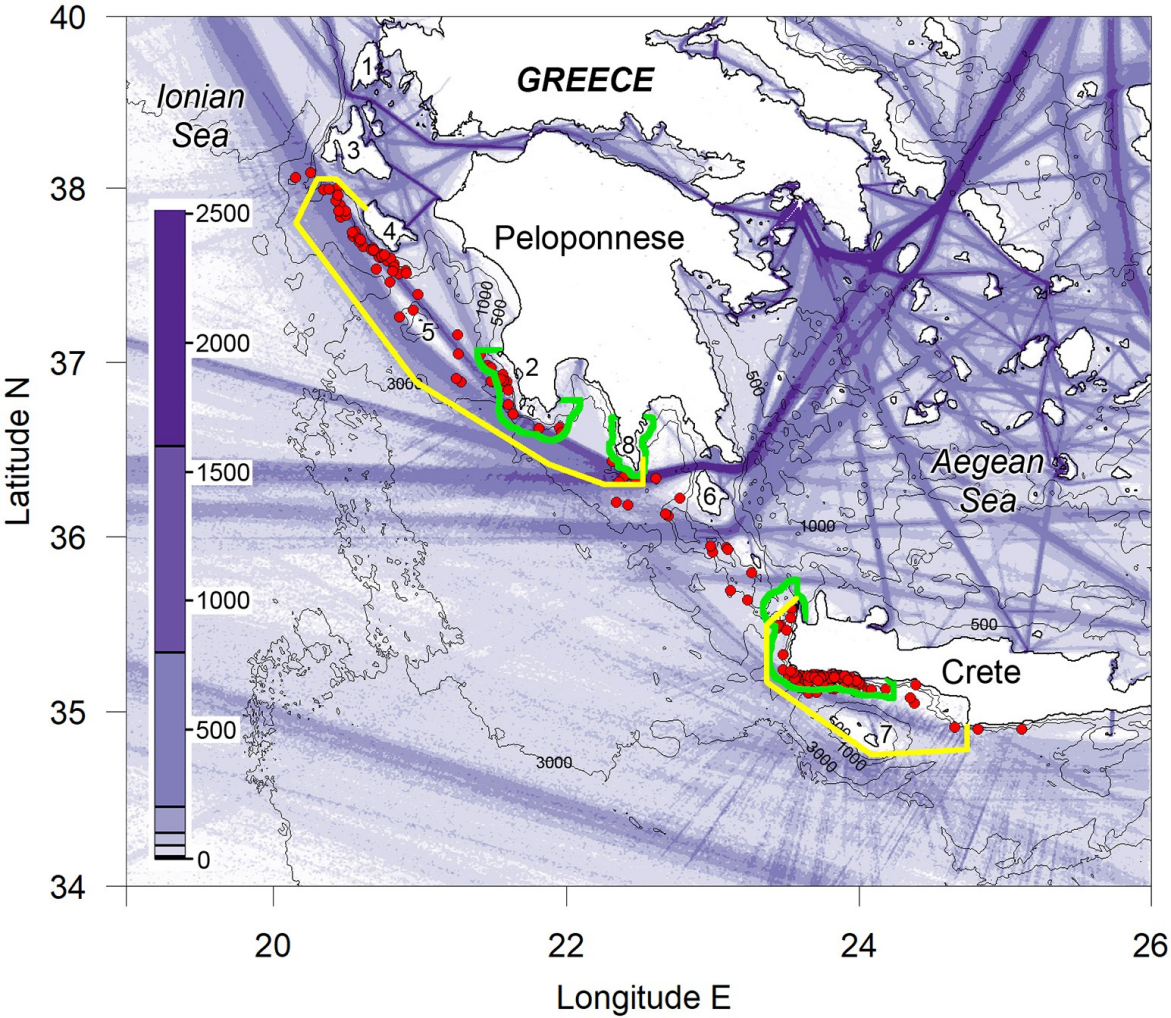
Possible routing options to reduce risks to sperm whales. Areas bounded by yellow lines would be avoided by vessels on the major routes. Density of ship traffic reported through AIS transmissions and sperm whale sightings (red dots) from our surveys as in Fig 5A. Areas bounded by green lines are the three legislated marine Natura2000 sites (Sites of Community Importance SCI) by the Greek Authorities in 2018 in order to protect cetacean habitat mainly. Numbers indicate Greek locations mentioned in the text as follows. 1: Lefkada Island, 2: Pylos, 3: Kefallonia Island, 4: Zakynthos Island, 5: Strofades Islands, 6: Kythira Island, 7: Gavdos Island, 8: Cape Tainaron.

The proposed routing system examined in this work is based on the joint principle of avoiding two areas (Fig 6) by major shipping routes and covering the minimum area that would still provide a high level of risk reduction. Thus, avoidance of the areas of important sperm whale habitat is achieved. The issue of reducing risk for the busiest channel between the Ionian and Aegean Seas south of Cape Tainaron (Location 8 on Fig 6) is difficult. The proposed shift south would keep traffic away from the 1000 m contour south of Cape Tainaron, but without introducing substantial course changes or additional distance for traffic passing through Elafonisos Strait north of Kythira Island (Location 6 on Fig 6). This traffic would also pass over the 1000 m contour west of Kythira Island at approximately the optimum angle of 90^o^. This would help to minimise the risk in that area, but it is difficult to quantify due to the complex bathymetry. Hence we have not presented any risk reduction for the Kythira Sea in Table 5. No measures are proposed between Kythira Island and Crete through Kythira or Antikythira Straits (between Kythira and Antikythira Islands and between Antikythira Island and Crete, respectively). Traffic passing through these channels has to cross sperm whale habitat but currently it does this at approximately 90^o^ and so risks are already minimised. These routes are also relatively little used by faster traffic such as Ro-Ro and container vessels. The relative risk indices for each zone with the current and alternative routing options that will avoid the two areas of sperm whale habitat as shown in Fig 6 are given in Table 5. The increased distance for the major routes are given in Table 6.

**Table 5:** Risk indices with the current and alternative (proposed) routing options that will avoid the two areas of sperm whale habitat as shown in Fig 6, together with the effort and data for each zone examined. The four zones are shown in Fig 1.

	Zakynthos Island and west Peloponnese (A)	Cape Tainaron (B)	Kythira Sea (C)	Crete (D)
Days with search effort	142	18	110	172
Days with sperm whales	86	3	10	85
Proportion of days	0.61	0.17	0.09	0.49
Total number of individual whales encountered	482	14	59	378
Mean shipping density (*D*_*sw*_) at whale locations (km^-1^year^-1^)	287.4	1581	332.7	66.6
Mean group size (G)	5.6	4.7	4.9	3.6
Collision risk index *C* (see Eq 2) in current situation	976	1230	179	146
Collision risk index *C* (see Eq 2) by re-routing	0	615	179	0

**Table 6:** Differences in distances for major routes with proposed new routeing options (nm stands for nautical miles).

Route	Original distance (nm)	Distance with proposed routeing measures (nm)	Increase (nm)
Suez Canal (Port Said) to Strait of Otranto	858.6	861.3	2.8
Westbound from southern tip of Peloponnese (Cape Tainaron) to Strait of Otranto, passing east of Strofades Islands	304.4	313.2	8.8
Eastbound from Strait of Otranto to southern tip of Peloponnese (Cape Tainaron), passing east of Strofades Islands	304.4	315.1	10.7
Westbound from southern tip of Peloponnese (Cape Tainaron) to Strait of Otranto, passing west of Strofades Islands	309.5	313.2	3.7
Eastbound from Strait of Otranto to southern tip of Peloponnese (Cape Tainaron), passing west of Strofades Islands	309.5	315.1	5.6

The maximum increase in distance is for traffic connecting the Adriatic and Aegean Seas, which currently passes east of Strofades Islands (37^o^ 14’ N, 21^o^ 00’ E; (Location 5 on Fig 6)). In this case, using a possible separation scheme routing further south of Cape Tainaron (Location 8 on Fig 6), the westbound traffic has to travel an extra 8.8 nautical miles and eastbound an extra 10.7 nautical miles (if these routes were using a Traffic Separation Scheme with a 2 nm wide Separation Zone). The choice of passage east of Strofades Islands (between Strofades Islands and the coast) is mainly made by Ro-Ro vessels. It is assumed that the 72% of container ships and 81% of passenger ships that choose to pass west of Strofades Islands do this for safety reasons. These vessels are already choosing a route that is 5.1 nautical miles longer than the passage closer inshore. This suggests that the increases in distance are of similar magnitude to decisions that are already taken on a regular basis by a large proportion of vessels.

There was some fishing by two or three vessels west of Zakynthos Island (Location 4 on Fig 5) in an area which would overlap with ship traffic transiting further offshore. However, this would only cover a small fraction of the current fishing area. Most of the fishing area is still likely to be to the west of the main shipping route. Apart from this there were no indications of concentrations of fishing vessels in offshore areas west of the Peloponnese and any routing changes are unlikely to have any negative impact. Coastal fisheries east of Strofades Islands (Location 6 on Fig 6) and off the SW tip of Crete currently overlap with shipping and overall safety would be improved by routing large vessels further offshore.

## Discussion

### Shipping density

The methods used here provided estimates of shipping density for areas of mixed satellite and terrestrial AIS coverage. In this study satellite data was available for 14% of the time. The median interval between satellite passes was 0.4 hours but the distribution had a long tail towards longer times. 5% of intervals were greater than 5 hours. Satellite data could have been used to reconstruct the track of a vessel assuming a straight line between received locations. This is the approach taken in other studies [19]. However, in areas of complex coastline and islands, it cannot be assumed that vessels will travel in a straight line between satellite passes, particularly over longer time intervals. The alternative approach used here is to allocate the distance travelled to the grid square in which the signal was received even though the travelling distance might take the vessel out of that grid square. This results in greater variance of the estimates for each grid square but avoids the problem of incorrectly assigning a track to a square the vessel did not pass through. The comparison of density estimates along a single route indicated that our methods showed no substantial bias with respect to the method of signal reception.

### The effect of a small shift offshore in shipping route

The current situation along the Hellenic Trench is that highly dense shipping routes run close along the 1000 m depth contour, which is the core of the localised habitat of a very small, endangered population. The evidence of collisions suggest that this situation is likely unsustainable for the sperm whale population when taking into consideration the size of the population [1] and the regular rate of strandings due to ship strikes or with signs of ship strikes. The main requirement to reduce ship strike risk for much of that habitat could be achieved in most areas by a small offshore shift in shipping route. This shift would add a maximum of 11 nautical miles to major routes and only around 5 nautical miles for the majority of ships. This is similar to the increase in distance that masters will regularly accept in routine precautionary routing decisions. There will nevertheless be mainly one area (west of Elafonisos Strait and northwest of Kythira Island; Location 6 on Fig 6) where re-routing is not an option due to the fact that ships have to cross almost perpendicularly the sperm whale habitat in order to head to or to exit through a narrow channel.

Beyond the increases in steaming distance, we have not identified any negative impacts associated with re-routing by shipping away from sperm whale habitat and there would be some additional safety benefits associated with traffic routing further from shore. IMO has adopted changes to seven Traffic Separation Schemes (TSS) and three Areas To Be Avoided (ATBA) since 2003 specifically for the purpose of reducing ship strike risks to cetaceans (measures implemented prior to 2010 are listed in Silber et al. [20] and the list is kept updated by IWC; see https://iwc.int/private/downloads/MgKVLOXOTu24cY--X55grQ/Ship_strike_reduction_measures_20170823.pdf). This process was further facilitated in 2009 by the adoption of the IMO guidance document [21]. The most recent changes relate to the TSS in the approach to the Panama Canal implemented in 2014 [22] and an ATBA off the coast of Costa Rica implemented in 2018 [23]. Both of these measures were designed to reduce risks for humpback whales. The only measure so far endorsed by IMO to reduce risks for sperm whales has been a seasonal, voluntary speed restriction to 13 knots in the Strait of Gibraltar established in 2007 [20]. In that case there were limited possibilities for any changes in routing because of the narrow confines of the Strait. Speed has been shown to be an important factor in ship strike risk with faster vessels being more likely to strike whales and with a greater chance of serious injury in the event of a collision [18]. Hence, based on the speed-risk relationship of Conn and Silber [18], Ro-Ro cargo ships, mainly taking lorries between Turkey and the northern Adriatic, present the highest risk by vessel type navigating along the Hellenic Trench. In other areas where routing options are not possible, speed reductions have been implemented. Restricting speed to 10 knots has been shown to be effective for reducing ship strike risk to North Atlantic right whales [18]. Speed restrictions could reduce risk from traffic passing in and out of the Aegean through the straits north and south of Kythira but would only contribute to a relatively small risk reduction compared to the routing options considered. In addition, routing measures are a widely accepted measure frequently implemented through IMO whereas speed restrictions are rare and have generally seen low compliance [20]. Thus speed restrictions were not considered within this study.

A similar situation with a high risk of ship-strikes to sperm whales occurs around the Canary Islands where the numbers of sperm whales around the islands was estimated at 224 individuals [24]. Based on demographic parameters in Whitehead [25], Fais et al. [24] suggested that the recruitment capability of this number of whales (224 individuals) is close to 2.5 whales per year, which was likely to be exceeded by the current ship-strike mortality rate in the Canary Islands. Frantzis et al. [1] suggested that the entire population that inhabits the Hellenic Trench is between 200 and 250 individuals and likely this is also the total for the entire Eastern Mediterranean Sea [26]. Although no recruitment capability has been estimated so far for the population along the Hellenic Trench, it would also be around 2.5 whales per year based on Whitehead [25]. The reported mortality rate from ship-strikes in the Greek Seas appears lower than in the Canary Islands (about one whale per year according to the data presented in Table 2). However, the actual number of ship-strike mortalities is underestimated by stranding data, since not all carcasses reach the coasts (some will eventually sink in the open sea; see [27]). Furthermore, the eastern Mediterranean social units of sperm whales are much more isolated and the Mediterranean population is isolated from its Atlantic conspecifics [26], ship strikes might occur in other places of the Eastern Mediterranean as well and the population unit of the Eastern Mediterranean also suffers mortality due to other anthropogenic causes (such as ingestion of plastic debris and entanglement in driftnets; [26]). As in the Canary Islands where ship-strikes appear to preferentially affect females and young animals [24], the Hellenic Trench is used mainly by social units (i.e. females, immature animals, juveniles and calves) [1] ship-strikes may increase the risk of population level effects.

In the studied case, the quantification of the total risk reduction to the population that might be achieved at the regional level after the application of the proposed routing measures is a difficult task. The average shipping density that whales in this area are exposed to (220 km^-1^year^-1^) is also among the highest in the Eastern Basin. For example the main route for global traffic between the Strait of Sicily and the Suez Canal was estimated at a similar density of 280 km^-1^year^-1^ in 2007 [17]. Outside the major routes, shipping densities are generally low. Sperm whales are known to move around the Eastern Mediterranean and have been encountered further offshore in the Ionian Sea [15], in the Aegean Sea [28], further east and south of the Hellenic Trench [28, 2], west and south of Cyprus [6] as well as east of Sicily [29]. However, in all other areas except the Hellenic Trench and potentially its short prolongation east in Antalya Bay, Turkey [30], the available knowledge indicates much lower densities [1, 2, 6]. If all shipping were to pass to the west of the two areas highlighted to be avoided in this study (Fig 6) then this would avoid all of the sperm whale high density areas and sighting locations from the surveys conducted in these areas [1]. Hence, at local level and, more specifically, in the core habitat for Eastern Mediterranean sperm whales, the overall reduction of the collision risk would be very high, varying from zero risk off Zakynthos Island (Location 4 on Fig 6) and off west Peloponnese, to half the current risk in Cape Tainaron (Location 8 on Fig 6), but with little risk reduction for the small area where the traffic has to pass through narrow straits around Kythira Island (Location 6 on Fig 6).

The densities of whales off Cape Tainaron are relatively low compared to elsewhere in the proposed avoidance areas, but shipping density is extremely high for the whales that do occur there. Thus, despite relatively low use by whales, this area does contribute substantially to the overall risk to the population (48% of the total risk). Based on the locations where whales have been observed in this area, the proposed shift in routing to the south would reduce risk by around 50%. The area between Kythira Island and Crete is the least used by whales of the zones considered in this study and only contributes about 7% to the estimated total risk. Based on the relative estimates of risk across the study area (Table 4), the proposed measures would reduce the overall collision risk for sperm whales in the study area by around 70%. In practice, some local traffic would still need to transit through the proposed areas to be avoided in order to serve local ports; however, this is most likely to be the smaller and slower vessels of the current traffic that pose lowest collision risk.

### Considerations for other important species, habitats and activities

Considerations related to reducing risk include any implications for other species that may be vulnerable to ship strikes and impacts on other human activities. The other large whale species that occurs in the Mediterranean and is known to be vulnerable to ship strikes [31, 32] is the fin whale (*Balaenoptera physalus*). Frantzis et al. [8] reviewed available data on fin whale distribution around the Hellenic Trench. Of 44 sightings of fin whales for all the Greek Seas for the period 1990–2014, the only area where fin whales had been observed repeatedly was in the northern Ionian Sea with a particular concentration north of Kefallonia (Location 3 on Fig 6) and west-northwest of Lefkada Islands (Location 1 on Fig 6). There were no records of fin whales in the sighting database from the area of the Hellenic Trench (including the coastal and offshore waters) from Kefallonia Island to Pylos (SW Peloponnese; Location 2 on Fig 6) or from West and SW Crete. This review led the IWC Scientific Committee to conclude that there is no reason to expect that routing measures designed to reduce risk to sperm whales would increase risk to fin whales [33].

The Hellenic Trench is also important habitat for Cuvier’s beaked whales (*Ziphius cavirostris*) [34, 28, 35], which is another deep diving cetacean species that is vulnerable to ship strikes. Although there are insufficient data on this species for a full risk analysis in our study area, Van Waerebeek et al. [36] reported on several likely Cuvier’s beaked whales ship strikes around the Canary Islands and also one case from New Zealand. Cuvier’s beaked whales have also been reported killed by ship strikes in Alaska [37] and off Sri Lanka [38]. The mean water depth and distance from the coasts of 63 Cuvier’s beaked whale sightings made along the Hellenic Trench was 1066 m and 8.6 km, respectively [28]. This closely coincides with the core habitat of sperm whales. Thus, the recently attributed status of IMMA in the area was entitled: “Hellenic Trench, Deep Diver's Sanctuary” in recognition of the importance to both species [5].

The Mediterranean sub-population of Cuvier’s beaked whales is classified as “Data Deficient” in the Red List of IUCN [39], but a proposal to change to “Vulnerable” is currently under review. The Hellenic Trench is the largest among five “High-density areas of occurrence” in the Mediterranean for Cuvier’s beaked whales [40] and very high observation rates have been recorded in the past [41]. However, the local population unit of Cuvier’s beaked whales has repeatedly suffered severe losses in four atypical mass stranding events (accounting for at least 45 individuals) due to military exercises of NATO and national navies that used military sonar [42, 34, 43, 40]. Mitigation of impacts due to shipping could also assist the recovery of Cuvier’s beaked whales.

Sea turtles are also vulnerable to collisions because they must surface to breathe [44]. The loggerhead turtle is another protected species (listed in Annex II species of EU Habitats Directive 92/43) inhabiting the studied area. The two most important nesting areas for this species in the entire Mediterranean Sea are the Laganas Bay in Zakynthos Island (Location 4 on Fig 6) and the Kyparissiakos Gulf in west Peloponnese [45]. Both sites are located along the coastal area of the Hellenic Trench and next to the current vessel traffic. In order to reach their breeding areas while coming from feeding grounds in other Mediterranean places and while heading back to feeding grounds, loggerhead have to cross the Hellenic Trench and the current vessel traffic. Shifting vessel traffic further offshore away from the sea-turtle high density areas is likely to reduce risks to sea turtles that congregate in coastal breeding grounds.

The effects of underwater noise from shipping are also likely to negatively impact all marine mammal species [46] present in the studied area. Apart the sperm whales, Cuvier’s beaked whales, striped dolphins and Risso’s dolphins inhabit the pelagic waters mainly above the slope of the continental shelf, while common bottlenose dolphins, the endangered short-beaked dolphins (Frantzis, 2009) and monk seals [47] inhabit the coastal waters. In 2002, the ACCOBAMS Parties adopted the Hellenic Trench as a potential pilot MPA, following the proposal of the ACCOBAMS Scientific Committee. The ACCOBAMS Scientific Committee re-asserted the urgency to create this MPA in November 2006 and, in early 2007 [4], refined the proposed boundaries [48]. The participants of a Global Scientific Workshop on Spatio-Temporal Management of Noise held in 2007 commended the ACCOBAMS proposal for an MPA covering most of the Hellenic Trench [48]. Recognizing that sperm whales and Cuvier's beaked whales inhabiting the Hellenic Trench are subjected to substantial noise level pollution from shipping traffic, military sonar, illegal dynamite fishing and increasing seismic survey activity, the Workshop Participants recommended shipping lane regulations to minimize the exposure of sensitive deep diving cetacean species to the cumulative high-intensity noise generated by maritime traffic. Increasing the separation between ships and the habitat of marine mammals in the area according to the proposed routing measures of this study will reduce the noise pollution effects to all these protected species.

The Workshop on Spatio-Temporal Management of Noise also recommended the designation by Greece of the SW Crete-Hellenic Trench MPA with a follow-up management plan to address threats to the species and the ecosystem. The Greek authorities have recently responded to the various international recommendations by officially including three large marine areas of the Hellenic Trench into the Natura2000 network (Fig 6) in January 2018. Although this is a positive step for cetacean conservation, the sperm whale density hot-spot off west Zakynthos Island (Location 4 on Fig 6) was left out of the network and the offshore borders of these Natura2000 areas cannot go further than six nautical miles (the area in Cape Tainaron extends only up to three nautical miles off the coasts; Location 8 on Fig 6), which is the limit of the declared national territorial waters of Greece (see Fig 6). The current Natura2000 areas do not include measures to address ship strikes or underwater noise pollution from ship traffic, but the routing measures considered in this study would benefit the pelagic cetacean species, as well as those inhabiting more coastal zones encompassed by the Natura2000 areas.

When proposing routing measures, navigation safety is a major issue that has to be taken into account. Fishing vessel presence may be considerably underestimated because vessels smaller than 15 m are not required to be equipped with AIS. However, the intention of the analysis of fishing vessel distribution in this study was to investigate whether changes in routing of larger commercial vessels further offshore would have any implications for fishing vessel safety. Fishing vessels less than 15 m and recreational vessels are more likely to be closer to the coast than fishing vessels greater than 15 m. Apart from the small area west of Zakynthos used by one or two fishing vessels, there was no indication that the routing options considered would have any impact on fishing activity and would be expected to reduce the overlap with smaller fishing and recreational vessels which were not equipped with AIS.

In conclusion, this study considered routing options that would most effectively reduce collision risk for sperm whales while minimising the inconvenience for the shipping industry. Among all possible options, only those that also provide a net improvement in overall maritime safety were considered. In particular, routing ship traffic further from vulnerable coastal areas was considered desirable where possible. The small routing changes proposed in the region of the present study would make a significant contribution to the overall conservation status of the Eastern Mediterranean sperm whale population and the Natura2000 sites in the area. There would also be additional environmental benefits if shipping avoided the suggested areas, including reduced impacts from shipping noise and reduced risk of any oil spills reaching the coasts, which include sensitive Natura2000 areas as well as important touristic destinations in Greece.

## Supporting information

S1 Fig **Acoustic listening stations (blue crosses) and visual sightings of sperm whales (red circles) of the surveys conducted in 2014, 2015 and 2017**.
(TIF)

S1 File Example of the raw AIS data files that amount in total ~11Gb and are available from the second author. (DOCX)

S2 File Processed AIS data in the grid form that was used to generate the main plots of shipping density. In each of the four sheets the first row is longitude and the first column is latitude for grid squares. Shipping density in sheets ‘A_AllShipDensityExceptFishing’, ‘B_ContainershipDensity’ and ‘C_RoRoShipDensity’ is given in km-1year-1. ‘D_FishingTime’ is given in days per year. The four sheets correspond to the A, B, C, D panels on Fig 5.
(XLSX)

S3 File Dates of eighty seven sperm whale sightings that originated from sources independent to our surveys. (XLS)

S1 Table Detection results for sperm whale year-round presence in the recordings of the Passive Acoustic Listener in Pylos. (XLSX)

S2 Table Sperm whale data used for the collision risk analysis. (XLSX)
