# Supplementary figures and images for: Shipping routes through core habitat of endangered sperm whales along the Hellenic Trench, Greece: Can we reduce collision risks?
Source: PLoS One. 2019 Feb 27;14(2):e0212016. doi: 10.1371/journal.pone.0212016 (PMC6392247; doi:10.1371/journal.pone.0212016)

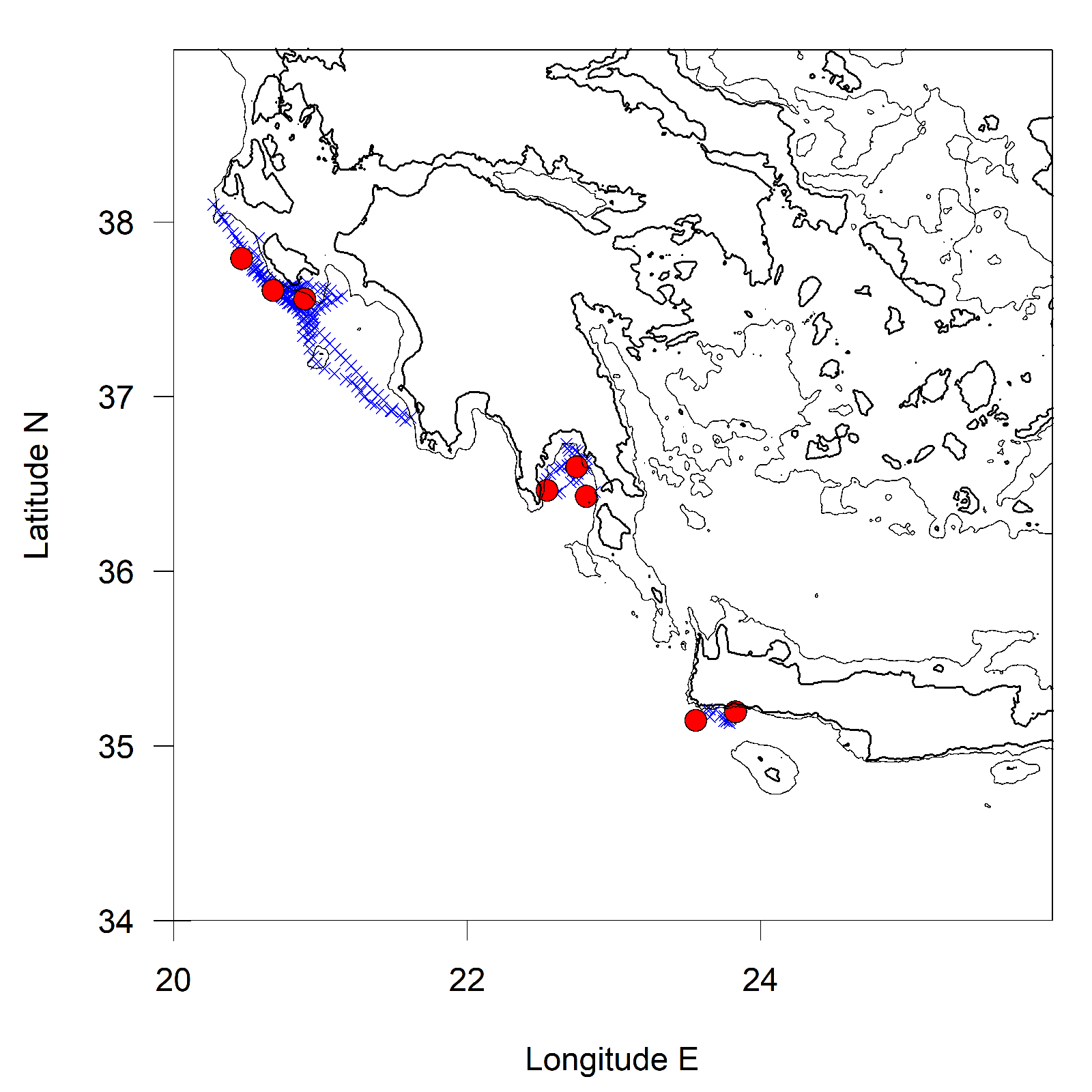

Supplement: S1 Fig — Acoustic listening stations (blue crosses) and visual sightings of sperm whales (red circles) of the surveys conducted in 2014, 2015 and 2017. (TIF) [file pone.0212016.s001.tif]
