# Supplementary material for: Shipping routes through core habitat of endangered sperm whales along the Hellenic Trench, Greece: Can we reduce collision risks?
Source: PLoS One. 2019 Feb 27;14(2):e0212016. doi: 10.1371/journal.pone.0212016 (PMC6392247; doi:10.1371/journal.pone.0212016)
Supplement: S1 File — (DOCX) [file pone.0212016.s002.docx]

| MMSI | SHIPTYPE | SPEED | COURSE | LON | LAT | TIMESTAMP_UTC | STATION |
| --- | --- | --- | --- | --- | --- | --- | --- |
| 309027000 | PASSENGERS SHIP | 154 | 298 | 22.57734 | 36.19878 | 01/06/2016 00:00 | TER |
| 241053000 | RO-RO CARGO | 140 | 345 | 23.95345 | 36.85501 | 01/06/2016 00:00 | TER |
| 215811000 | CRUDE OIL TANKER | 119 | 326 | 22.46447 | 35.97585 | 01/06/2016 00:00 | TER |
| 247282900 | PASSENGERS SHIP | 180 | 116 | 21.77305 | 36.61869 | 01/06/2016 00:00 | TER |
| 538005617 | VEHICLES CARRIER | 166 | 104 | 19.1139 | 35.20609 | 01/06/2016 00:00 | TER |
| 477006500 | CONTAINER SHIP | 150 | 94 | 19.83667 | 36.40227 | 01/06/2016 00:00 | TER |
| 209587000 | RESEARCH/SURVEY VESSEL | 112 | 284 | 18.11745 | 35.2776 | 01/06/2016 00:00 | TER |
| … |  |  |  |  |  |  |  |
